# A Call for Complexity in the Study of Social Anxiety Disorder. Commentary: The aetiology and maintenance of social anxiety disorder: A synthesis of complementary theoretical models and formulation of a new integrated model

**DOI:** 10.3389/fpsyg.2016.01963

**Published:** 2016-12-20

**Authors:** Alexandre Heeren, Richard J. McNally

**Affiliations:** ^1^Department of Psychology, Harvard UniversityCambridge, MA, USA; ^2^Psychological Science Research Institute, Université Catholique de LouvainLouvain-la-Neuve, Belgium

**Keywords:** social anxiety disorder, computational social science, network analysis, complexity, graph theory, etiological factors, maintenance factors, experimental psychopathology

Wong and Rapee (2016) conducted a much-needed comprehensive review of etiological and maintenance models of social anxiety disorder (SAD) to formulate a cutting-edge integrative model. We agree that the threat value assigned to social-evaluative stimuli may act as core process bridging etiological (e.g., peer rejection) and maintenance (e.g., attentional bias for threat) factors of SAD that eliminate (e.g., avoidance) potential threat. Their model persuasively postulates multiple causal pathways and loops whereby variables increasingly reinforce the threat-value of social-evaluative stimuli so that they foster the development of secondary processes to further detect and reduce potential threat, culminating in full-blown SAD. We believe that the computational and conceptual tools of network analysis (Borsboom and Cramer, 2013) can render testable the complex dynamic features of their model.

During the last decade, network science has transformed disciplines such as ecology, physics, and sociology (Barabási, 2012). With the recent advances of Borsboom and his colleagues at both the theoretical (Borsboom and Cramer, 2013) and computational levels (Epskamp et al., 2012), we are entering the period when this “network takeover” (Barabási, 2012, p. 14) is opening up new vistas for understanding psychopathology (McNally, 2016; Borsboom, in press). At the simplest level, a network consists of nodes and edges that connect them. In psychopathology, nodes represent symptoms and edges represent association between symptoms. The network approach conceptualizes an episode of disorder as emerging from the dynamic interplay of symptoms. Symptoms possess independent causal powers that influence other symptoms; they are not merely passive indicators of an underlying disease. Hence, symptoms are constitutive, not reflective, of disorder (Borsboom and Cramer, 2013).

Of critical importance, network approach allows examining the extent to which nodes are central to the network based on the amount and direction of influence that flows from one node to other ones. Activation issuing from a highly central symptom can thus spread to other symptoms, thereby producing a full-blown episode of disorder. Given how Wong and Rapee postulates multiple maintenance factors whose persistence occurs via feedback loops among them, we believe that viewing these factors as intertwined nodes of a network, whose edges represent the association among them, can illuminate how these factors interact as whole system, causing and maintaining SAD.

Although evidence supports the associations of each of Wong and Rapee's factors with SAD, knowledge about the precise wiring of the model's pieces via the computational methods of network analysis would allow ranking the maintenance factors based on their level of centrality or influence within the entire network; a pivotal phase that may ultimately lead to the identification of factors that can trigger other ones, thereby propagating activation through the whole network and maintaining the disorder. Particularly, the authors postulate multiple contributing pathways and loops whereby variables increasingly reinforce the threat-value assigned to social-evaluative stimuli. Consequently, threatening social-evaluative stimuli should exhibit the highest level of centrality within the entire network. Likewise, Wong and Rapee hypothesize that the central role of the threat-value that is assigned to social-evaluative stimuli is reflected at the neurobiological level by amygdala activity, and especially dysfunctional connectivity between the amygdalae and the frontal areas. In this way, the application of network analysis over joint neuroimaging and laboratory-based measurements would allow to explore whether brain network does mimic psychological network, with the amygdala and the threat-value that is assigned to social-evaluative stimuli respectively acting as central hubs.

Wong and Rapee hold that interactions among the maintenance factors lead to full-blown SAD. Network analysis can test precisely how these processes unfold. For example, in addition to symptom reports, one can include laboratory measures tapping attentional bias for threat, executive control over attention, and so forth within the same computational process. For example, we recently demonstrated how avoidance of social situations and the orienting component of attention both act as core hubs within the entire network of SAD symptoms, attentional bias for threat, and measures of attentional control among patients with SAD diagnosis (Heeren and McNally, 2016).

Finally, Wong and Rapee also postulate that several etiological factors, such as bullying, culture, or inherited tendencies, influence how people assign threat-value to social-evaluative stimuli. Although traditional longitudinal studies allow tracking variables over time, recently developed computational methods allow exploring the within- and between-person temporal dynamics of networks (Epskamp et al., 2016a). In this way, such an approach may provide tools capable of testing whether the network trajectory vary across individuals with SAD so that the temporal dynamic interplay among the etiological factors conspire to transform the threat-value that is assigned to social-evaluative stimuli into a central hub among the network of maintenance factors. Moreover, as some etiological factors are stable (e.g., culture), techniques from network comparison (e.g., van Borkulo et al., 2015) may also help to identify the impact of a given etiological factor on the network dynamics. For instance, as the model assume that aspects of an individual's culture influence the interactions among the maintenance factors by foster the “centrality” of threat-value assigned to social-evaluative stimuli, comparing the network dynamics of individuals from Western countries to Asian countries would allow directly testing this assumption.

In many applications of network analysis, one need not estimate parameters. For example, to compute a network illustrating collaboration among scientists, one can directly ascertain whether two scientists have co-written one or more articles; one need not “estimate” whether they have published together. This does not hold for networks illustrating symptom-symptom connections; one must estimate these parameters (Epskamp et al., 2016b). To do so reliably requires many subjects when the number of parameter estimates is large (e.g., 362 subjects relative to 17 symptoms; McNally et al., 2015). Unfortunately, to integrate laboratory measures into network analysis can prove challenging as few experimental psychopathology studies have more than 30 subjects per group. Fortunately, statisticians have devised procedures that render tractable such high-dimensionality problems (Friedman et al., 2008). Yet uncertainty remains about the optimal way to estimate networks comprising cognitive, behavioral, and biological processes other than assessment of self-reported symptoms. Indeed, such studies are rare (Heeren and McNally, 2016; Hoorelbeke et al., 2016).

## Author contributions

AH had the initial ideas and wrote the first draft of the manuscript. All authors then revised the manuscript critically and contributed to and have approved the final manuscript.

### Conflict of interest statement

The authors declare that the research was conducted in the absence of any commercial or financial relationships that could be construed as a potential conflict of interest.
